# Is a diet low in greenhouse gas emissions a nutritious diet? – Analyses of self-selected diets in the LifeGene study

**DOI:** 10.1186/s13690-017-0185-9

**Published:** 2017-04-10

**Authors:** Katarina Bälter, Camilla Sjörs, Arvid Sjölander, Christopher Gardner, Fredrik Hedenus, Annika Tillander

**Affiliations:** 1 Department of Medical Epidemiology and Biostatistics, Karolinska Institutet, Nobels väg 12a, 171 77 Stockholm, Sweden; 2 Stanford Prevention Research Center, Stanford School of Medicine, Stanford, USA; 3 Division of Public Health Sciences, School of Health, Care and Social Welfare, Mälardalen University, Västerås, Sweden; 4 Department of Energy and Environment, Chalmers University of Technology, Gothenburg, Sweden

**Keywords:** Diet, Nutrients, Carbon dioxide equivalents, Greenhouse gas emission, Life cycle assessment, Nordic Nutrition Recommendations, LifeGene study

## Abstract

**Background:**

Climate change is an urgent global issue and the food sector is a major contributor to greenhouse gas emissions (GHGE). Here we study if a diet low in GHGE could be a nutritious diet compared to the Nordic Nutrition Recommendations (NNR).

**Methods:**

The environmental impact of foods from Life Cycle Assessment (LCA) data was linked to a food frequency questionnaire (FFQ) filled out by 5,364 participants in the Swedish LifeGene study. Thereafter, we calculated the daily emission of CO_2_ equivalents (CO_2_e) as well as the intake of selected nutrients associated with vegetables, fruits, meat and dairy products. The CO_2_e was divided into quartiles were quartile 1 corresponds to a diet generating the lowest CO_2_e, and quartile 4 corresponds to a diet with the highest CO_2_e.

**Results:**

The overall diet-related emission was 4.7 kg CO_2_e/day and person, corresponding to 1.7 ton CO_2_e/year. In general, there were only small differences in nutrient intake between groups of varying levels of CO_2_e, regardless if the intake was analyzed as absolute intake, energy percent or as nutrient density. Moreover, adherence to NNR was high for the group with the lowest CO_2_e, except for saturated fat where the intake was higher than recommended for all CO_2_e groups. On the other hand, only the group with the lowest CO_2_e fulfilled recommended intake of fiber. However, none of the CO_2_e groups reached the recommended intake of folate and vitamin D.

**Conclusions:**

Here we show that a self-selected diet low in CO_2_e provides comparable intake of nutrients as a diet high in in CO_2_e.

**Electronic supplementary material:**

The online version of this article (doi:10.1186/s13690-017-0185-9) contains supplementary material, which is available to authorized users.

## Background

Substantial reductions of greenhouse gas emissions (GHGE) are needed if the global warming should be limited to the UN target of a maximum of 2 °C, compared to pre-industrial times, and dietary shifts towards a more climate friendly diet are one of several strategies to reduce emissions from the food sector [1–3]. However, when promoting a diet low in GHGE, it is important to consider health aspects of the diet and adherence to dietary recommendations. Previous studies based on simulated dietary scenarios have reported that a diet low in GHGE can be nutritious at the same time [2, 4, 5], but among studies based on self-selected diets [6–9], only one reported GHGE in relation to the intake of macronutrients and fiber [10]. Therefore, more research is needed to study intake of vitamins, minerals, macronutrients and fiber among individuals with a diet low in GHGE [11].

The production of food is estimated to contribute to 25% of the total GHGE in the world, more than the total emissions from the transport sector [12]. However, there is a substantial variation in GHGE for different food products and the production of food items from animal origin is generally associated with higher GHGE than plant-based food items, such as vegetables, whole grains and legumes. Particularly, meat from ruminants, such as cattle and sheep, are associated with high emissions due to their less efficient feed-conversion rate and to the methane produced during digestion [13, 14]. Our collective food choices have significant impact on global GHGE and a dietary shift may significantly impact public health.

Here we estimate diet-related GHGE in the Swedish LifeGene study using a Food Frequency Questionnaire (FFQ) linked to Life Cycle Assessment (LCA) data of carbon footprint for food products representative for Swedish food habits. The overall aim is to investigate if a diet low in GHGE can be a nutritious diet. To the best of our knowledge this is the first Swedish study reporting the intake of several nutrients from a self-selected diet low vs. high in GHGE. Second, it is the first study relating GHGE to the Nordic Nutritional Recommendations (NNR) [15] on an individual level for nutrients associated with meat, dairy, fruits and vegetables.

## Methods

The LifeGene study is a prospective Swedish cohort study aiming at combining advances in modern biotechnology with information on individual’s health and lifestyle [16]. The target enrollment in LifeGene is 300,000 Swedes, with the projected follow up of 20 years [17]. The present study is based on the pilot phase of the LifeGene study, which was launched in Stockholm in October 2009, followed by Umeå in November and Alingsås in January 2010, and invitations were sent out until March 31, 2010. In total, 42,700 women and men age 18–45 years old were randomly selected through the national population registry and invited to the study. An invitation letter with personal login information was sent out, including up to three reminders. After the study participants agreed to participate and left consent on the LifeGene web page, they were asked to respond to a comprehensive interactive web questionnaire at home, including questions on lifestyle factors, self-care, women’s/men’s health, living habits, health history, injuries, asthma and allergy, mental health, home and work. Thereafter, an appointment at one of the test centers was made for in-person testing. 7,818 filled out part of, or the whole questionnaire, and 6,633 visited the test center. The Research Ethics Review Board at Karolinska Institutet approved the present study.

### Dietary assessment method

Diet was assessed using the interactive web-and meal based FFQ called Meal-Q, described in detail elsewhere [18, 19]. Meal-Q assesses habitual dietary intake during the previous months and includes 102–174 food items, dishes, and beverages, depending on the number of follow-up questions, as well as questions about supplement use, meal patterns, and eating behavior. Participants were instructed to choose among predefined food items and intake frequencies and report on all items that were consumed at least once a month. Five photos of different portion sizes were included for 1) rice, potatoes and pasta, 2) meat, chicken, fish and vegetarian substitutes and 3) vegetables (raw or cooked) and used to calculate portion sizes for cooked dishes and vegetables whereas a standard portion sizes were used for all other food items. A program tailored for Meal-Q called NutriCalc was used to link dietary data to the national food composition table from the Swedish National Food Agency [20] to generate the daily intake of energy and nutrient per person. Meal-Q has been validated using 7-day weighed food records with regards to nutrients and energy as well as doubly labeled water with regards to energy, demonstrating good validity and reproducibility [18, 19].

### Diet-related GHGE

We identified published LCA data for 65 food items and food groups representative of typical food consumption in Sweden described in detailed elsewhere and about half of the LCA data came from the same source [21]. These 65 food items and groups matched the food items/questions in Meal-Q, for example, we asked for 3 kinds of bread in Meal-Q (white, whole grain and crisp, respectively), but applied the same LCA data for all 3 kinds of bread. Assessment of the combined impact of different greenhouse gases was achieved using Global Warming Potential (GWP) with a 100 years perspective expressed as kg carbon dioxide equivalents (CO_2_e) per kg of food product. The GWP used to calculate the CO_2_e was 1 for carbon dioxide, 34 for methane and 296 for nitrous oxide [22].

The GHGE include emissions from agriculture and its inputs, food processing, distribution and retailing [21]. If LCA studies did not include emissions from distribution and retailing, emission were imputed by adding emissions linked to retail, transportation and packaging using Swedish data [21]. Emissions after the retail phase were not included, such as transports to the household, storing and cooking, as well as from waste management.

Portion sizes in Meal-Q were based on food ready to be eaten and therefore we recalculated LCA data for uncooked food to CO_2_e per kg cooked food when needed, considering both hydration, i.e. cooking of rice, and dehydration, i.e. cooking of meat [21]. In addition, we adjusted for unavoidable food losses (i.e. shell and bone) using data from the Swedish food composition database [20] and avoidable food waste both before and after food preparation using data from the British Waste and Resources Action Programme [23] and a FAO report [24].

CO_2_e for mixed dishes was based on up to three main food products or groups and weighed using standard recipes from the Swedish food composition database [20], for example, lasagna was based on weighted LCA data from ground meat, milk and tomato, respectively. Thereafter, data on CO_2_e per kg food item were linked to all food items in Meal-Q by the NutriCalc program to calculate daily CO_2_e per person. The assessment of CO_2_e by Meal-Q was validated using 7-day weighed food records and the Spearman correlation coefficient between CO_2_e from Meal-Q and the 7-day weighed food records were *r* = 0.70 (95% CI 0.61–0.77), whereas 90% were categorized into the same/adjacent quartile in cross-classification analyses [21]. The intraclass correlation coefficient for the reproducibility of Meal-Q was 0.81(95% CI 0.73–0.87), and 94% were categorized into the same/adjacent quartile in cross-classification analyses [21].

### Nordic nutritional recommendations (NNR)

The main goal for the NNR is to set guidelines to promote good health and to prevent major chronic diseases in the population in the Nordic countries [15]. The NNR includes recommendations regarding intake of nutrients; total energy intake, intake of macronutrients as a percentage of total energy intake, intake of fiber and salt, as well as recommended daily intake of vitamins and minerals. Selected recommendations of relevance for the present study are described in Table 1.

**Table 1:** Description of the recommendations in the Nordic Nutrition Recommendations (NNR) from 2012

	Recommended intake^a^		Goal for menu planning^b^
	Women	Men	Nutrient/MJ
Energy (kJ)^c^	8,500	11,000	
Protein (E%)^d^	10–20	10–20	
Carbohydrates (E%)^d^	45–60	45–60	
Fat (E%)^d^	25–40	25–40	
Saturated fat (E%)^d^	<10	<10	
Monounsaturated fat (E%)^d^	10–20	10–20	
Polyunsaturated fat (E%)^d^	5–10	5–10	
β-carotene (μg)^e^	-	-	-
Vitamin C (mg)	75	75	8
Folate (μg)	300/400^f^	300	45
Fiber (g)	25–35	25–35	3
Vitamin B12 (μg)	2.0	2.0	0.2
Iron (mg)	9/15^g^	9	1.6
Zinc (mg)	7	9	1.1
Vitamin D (μg)	10	10	1.3
Retinol (μg)^e^	-	.	.
Retinol equivalents (RE)^h^	700	900	80
Calcium (mg)	800	800	100

^a^Recommended intake for women and men age 18–60 years

^b^Goals for menu planning expressed as nutrients/MJ for age 6–65 taking into account sub-groups with the highest nutrient requirements in the population

^c^Reference values for a person age 31–60 with a BMI of 23 with sedentary work

^d^Not including energy from alcohol. 1 gram of fat = 37 kJ, 1 gram of protein = 17 kJ, 1 gram of carbohydrate = 17 kJ

^e^No value determined

^f^Women in child-bearing age

^g^Menstruating women

^h^1 Retinol equivalents (RE) = 1 μg retinol = 12 μg β-carotene

### Test centers

The in-person clinical testing included measurements of weight, height, waist, hip and chest circumference, bioimpedance, heart rate and blood pressure along with audiometry and spirometer. Blood and urine samples were taken for analyses and biobanking.

### Statistical analysis

Participants with energy intake less than 3,300 or more than 21,000 kJ were excluded (*n* = 212). The purpose of the cut-off is to exclude participants with implausibly high or low total calorie intake, thus, improving the quality of the data that is being analyzed. Quartiles were used to split CO_2_e into four groups, both for crude values and energy adjusted values using the residual method [25] and quartile 1 corresponds to the group with the lowest CO_2_e, and quartile 4 to the group with the highest CO_2_e. Median and interquartile range (25^th^–75^th^ percentile) of the nutrient intake divided by CO_2_e groups was calculated and the difference tested with Kruskal-Wallis test. To show the distribution within the energy adjusted CO_2_e groups, the nutrients are presented as boxplots in Figs. 2 and 3. The notch corresponds to the median, the edges of the box correspond to the first quartile (*q*_1_) and third quartile (*q*_3_). The vertical lines at the end of the dotted line are the lower and upper adjacent value (LAV and UAV) here calculated as follows: LAV = smallest value which is ≥ *q*_1_−4 *IOR*; UAV = largest value which is ≤ *q*_3_ + 4 *IOR*, where IQR is the interquartile range (*IOR* = *q*_3_−*q*_1_). Due to large sample size we chosen 4 instead of commonly used 1.5 to highlight extreme observations to make more distinguishable graphs. The extreme observations, values below the LAV or above UAV, are marked as circles. All analyses were performed in the statistical software STATA version 13.1. Significance level was set to α = 0.05.

## Results

In total, 5,576 participants filled out the section about diet in the questionnaire, of which 5,364 also visited the test center. Table 2 shows the characteristics of the study participants. The majority of participants had a normal BMI of <25 kg/m^2^, had more than a high school education and the overall median age was 32 years. The crude median diet-related emission was 4.7 kg CO_2_e/day and person, corresponding to 1.7 ton CO_2_e/year, and the median emissions were lower for women, 4.4 kg CO_2_e/day and person, than for men, 5.3 kg CO_2_e/day. 23% and 22% of the women and men, respectably, reported using multivitamin and/or mineral supplements. The consumption of beef (including ground meat and hamburgers) was 0.3 servings per day for women and 0.5 for men which correspond to 2.1 and 3.5 servings per week, respectively, whereas the consumption of all types of meat was 0.9 and 1.0 per day or 6.3 and 7 servings per week for women and men, respectively. Additional data on nutrient intake is shown in Additional file 1: Table S1.

**Table 2:** Characteristics of the participants in the Swedish LifeGene study in 2009–10

Characteristics	Women		Men		All	
	(*n* = 3,239)		(*n* = 2,125)		(*n* = 5,364)	
	Median	(IQR)	Median	(IQR)	Median	(IQR)
CO_2_e (kg/d)	4.4	2.0	5.3	2.3	4.7	2.2
Age (years)	32	14	34	12	32	12
BMI (kg/m^2^)	22.9	4.2	24.8	4.1	23.7	4.5
Servings of beef/day^a^	0.3	0.3	0.5	0.3	0.3	0.3
Servings of meat/day^b^	0.9	0.5	1.0	0.5	1.0	0.6
Servings of dairy products/day^c^	1.1	1.2	1.2	1.5	1.1	1.4
Servings of dairy products and dishes/day^d^	1.2	1.2	1.3	1.5	1.2	1.4
	N	%^e^	n	%^e^	n	%^e^
Education (years)^e^						
< 9	44	1.4	39	1.9	83	1.6
9–12	757	23.5	614	29.2	1,371	25.8
> 12	2,134	66.3	1,286	61.2	3,420	64.3
Other	283	8.8	162	7.7	445	8.4
Tobacco users^f^	422	13.2	455	21.8	877	16.6
Supplement use^g^	757	23.5	463	21.9	1,220	22,.9

^a^Beef, hamburgers and ground meat dishes

^b^Beef, hamburgers, ground meat dishes, pork, bacon, lamb, game, offal, chicken

^c^Milk, yoghurt, hot cocoa, cheese (hard and soft), ice cream

^d^Milk, yoghurt, hot cocoa, cheese (hard and soft), ice cream, pancake, pizza

^e^Percentages are averaged why their sum may exceed or not reach 100%

^f^Current smoking and/or snuff use

^g^Users of multivitamin and mineral supplement

**Fig. 1:**
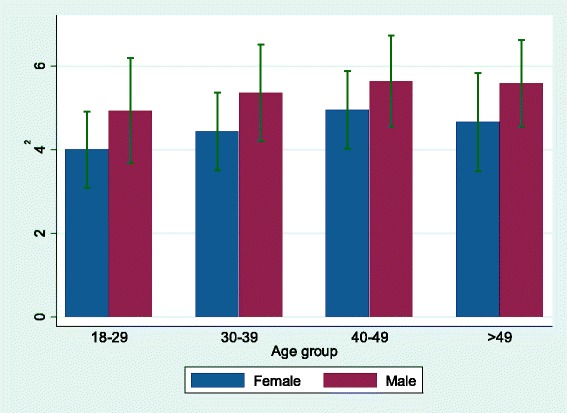
Diet-related greenhouse gas emissions by age and gender in the LifeGene study, 2009–10, Sweden (*n* = 5,364). The distribution of crude median and IQR of daily diet-related greenhouse gas emissions expressed as kg CO_2_e by age and gender

The distribution of crude daily CO_2_e by age and gender is shown in Fig. 1. The median CO_2_e was lower in women than in men, and increased with age for both women and men. The lower CO_2_e in women compared to men is an effect of lower general intake of energy in women as well as gender differences in what type of food they eat (i.e. higher meat intake in men). Therefore, to take into account differences in energy intake, we present the median and interquartile range (IQR) of absolute nutrient intake according to quartiles of energy adjusted CO_2_e in Table 3. The intake of nutrients mainly coming from plant-based foods, such as β-carotene, carbohydrates, polyunsaturated fat, and fiber, were higher in the group with the lowest CO_2_e compared to the group with highest CO_2_e, except for the intake of monounsaturated fat, vitamin C and folate that was higher in the highest CO_2_e group. Nutrients serving as markers for intake of meat and dairy, such as vitamin B_12_, zinc, vitamin D, retinol equivalents, calcium, fat, saturated total fat, and protein, were generally higher in the highest CO_2_e group, compared to the lowest CO_2_e group, whereas there were only small differences regarding the iron intake between CO_2_e groups. The overall result did not change when CO_2_e was divided into three groups, i.e. tertiles as well as five groups i.e. quintiles, see Additional file 2: Table S2 and Additional file 3: Table S3.

**Table 3:** Median nutrient intake according to daily diet-related greenhouse gas emissions in the LifeGene study, 2009–10, Sweden

	kg CO_2_e/d				
Nutrients	0.2–<4.1	4.1–<4.8	4.8–<5.7	5.7–10.9	*P*-value^1^
	Median (IQR)	Median (IQR)	Median (IQR)	Median (IQR)	
Energy (kJ/d)	8432 (4064)	7396 (2995)	8086 (3081)	8700 (3413)	< 0.00
Protein (g/d)	71.1 (35.8)	72.3 (28.2)	76.8 (30.6)	87.6 (34.9)	< 0.00
Carbohydrates (g/d)	243.1 (125.2)	215.0 (95.6)	215. 9 (97.6)	219.1 (102.2)	< 0.00
Fat (g/d)	64.8 (37.3)	62.6 (27.9)	65.1 (28.8)	70.5 (32.5)	< 0.00
Saturated fat (g/d)	24.0 (14.9)	23.5 (11.6)	24.7 (12.6)	27.2 (13.1)	< 0.00
Monounsaturated fat (g/d)	23.9 (13.4)	23.1 (10.5)	24.1 (11.2)	26.1 (12.1)	< 0.00
Polyunsaturated fat (g/d)	11.3 (7.3)	10.4 (5.6)	10.6 (5.7)	10.7 (5.6)	< 0.00
β-carotene (μg/d)	2,514 (2,766)	2,317 (2,350)	2,445 (2,440)	2,481 (2,326)	0.07
Vitamin C (mg/d)	92.0 (71.2)	95.5 (66.9)	99.3 (71.3)	103.9 (75.6)	< 0.00
Folate (μg/d)^2^	296.4 (192.2)	287.5 (143.5)	291.3 (140.1)	302. 3 (146.8)	< 0.00
Fiber (g/d)	24.8 (18.0)	22.2 (13.2)	21.7 (11.6)	21.1 (11.5)	< 0.00
Vitamin B12 (μg/d)	3.7 (2.6)	4.3 (2.2)	4.8 (2.3)	5.8 (2.9)	< 0.00
Iron (mg/d)^3^	13.4 (8.3)	12.5 (6.4)	12.9 (6.1)	13.6 (6.2)	< 0.00
Zinc (mg/d)	9.7 (5.2)	9.7 (4.0)	10.3 (4.2)	11.7 (4.6)	< 0.00
Vitamin D (μg/d)	4.8 (3.7)	5.4 (3.2)	5.9 (3.1)	7.0 (3.8)	< 0.00
Retinol (μg/d)	364.8 (310.3)	388.2 (270.9)	423.8 (254.6)	464.7 (303.2)	< 0.00
Retinol equivalents (RE/d)	674.4 (450.1)	652.5 (392.5)	685.2 (386.8)	724.3 (426.5)	< 0.00
Calcium (mg/d)	816.4 (511.3)	849.6 (443.4)	947.4 (457.9)	1,055.5 (574.0)	< 0.00

Median and interquartile range (IQR) of nutrient intake according daily diet-related greenhouse gas emissions (CO_2_e) adjusted for total energy intake among 5,364 men and women in the LifeGene study

^1^Kruskal-Wallis p-values

^2^For women only: the intake of folate was 301.7, 295.4, 297.4, 310.8 μg/d for varying levels of kg CO_2_e/d

^3^For women only: the intake of iron was 13.1, 12.5, 12.5, 13.2 mg/d for varying levels of kg CO_2_e/d

**Fig. 2:**
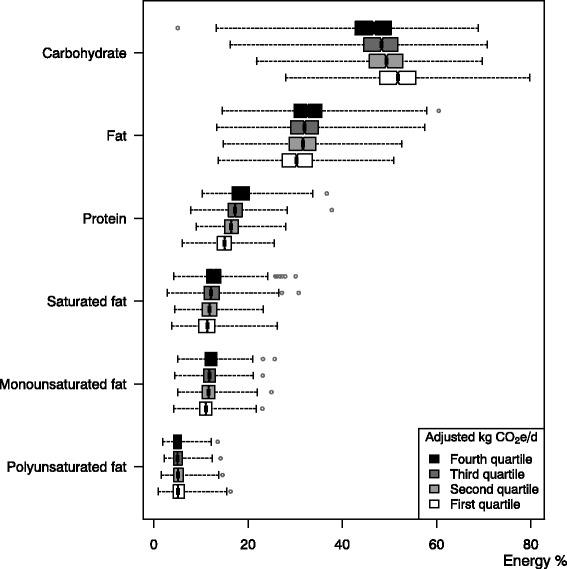
Percentage of energy coming from macronutrients by quartiles of increasing levels of greenhouse gas emissions in the LifeGene study, 2009–10, Sweden (*n* = 5,364). The percentage of energy coming from fat, protein, carbohydrates, saturated fat, monounsaturated fat, and polyunsaturated fat, respectively, by quartiles of increasing levels of energy adjusted diet-related greenhouse gas emissions

Figure 2 show the percentage of energy coming from fat, protein, carbohydrates, saturated fat, monounsaturated fat, and polyunsaturated fat, respectively, by quartiles of increasing levels of diet-related CO_2_e. Overall, there were small differences between quartiles of CO_2_e and the energy percent for the different macronutrients were in line with recommended intake according to NNR described in Table 1, except for saturated fat which was higher than recommended for all CO_2_e groups.

**Fig. 3:**
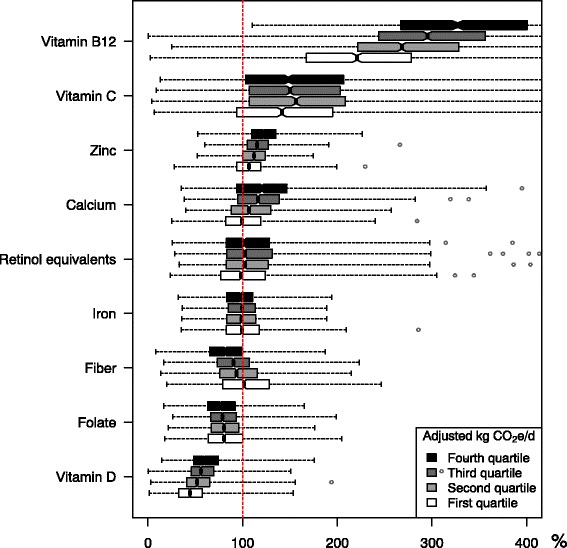
The nutrient density (nutrient/MJ) by quartiles of increasing levels of greenhouse gas emissions in the LifeGene study, 2009–10, Sweden (*n* = 5,364). The nutrient density (nutrient/MJ) by quartiles of increasing levels of energy adjusted diet-related greenhouse gas emissions. Hundred percent corresponds to nutrient density goals for menu planning according to the Nordic Nutrition Recommendation

Figure 3 shows the nutrient density (nutrient/MJ) by quartiles of increasing levels of diet-related CO_2_e. Hundred percent corresponds to goals for menu planning according to the NNR. Again, there were small differences for vitamins, minerals and fiber between quartiles of CO_2_e, except for vitamin B_12_, where all groups substantially exceeded the recommended intake of B_12_. In contrast, none of the groups reached the recommended intake of folate and vitamin D. Moreover, for fiber, only the group with the lowest CO_2_e reached recommended intake.

## Discussion

The group with the lowest CO_2_e emissions had adequate intake of most nutrients, and the intake was comparable with the nutrient intake among people in the highest CO_2_e emissions, suggesting that a diet low in GHGE can be nutritious at the same time. Although the intake of some important nutrients increased with increasing emissions in our study, there were only small differences between groups with varying CO_2_e levels and the effect was less pronounced compared to a study by Vieux et al 2013 where an indicator of good nutritional quality dietary was significantly higher for a diet high in GHGE compared to a diet low in GHGE [9].

Compared to the NNR [15], the median intake of micronutrients was generally near or above the recommended intakes when analyzed as nutrient density in all CO_2_e groups in the present study. The trend was similar for energy percent of macronutrients, where the intake was within recommended levels, except for saturated fat which was higher than recommended. In general, all CO2e groups fulfilled the recommended intake of iron, B12, zinc, retinol equivalents, nutrients mainly found in animal products. Highest intake of B12 and zinc were found in the group with the highest CO_2_e, whereas there were no differences for iron and retinol equivalents. However, the intake of folate did not reach the recommended level for women in reproductive age, which is a majority of the females in this study. Nor did any of the groups have enough intake of vitamin D, a common problem in the general population in Sweden.

This study has several strengths and limitations that should be considered. A main strength is the use of individually assessed dietary information from a large population-based study, which allows for analyses of individual variability in CO_2_e, whereas many studies in this area rely on simulated dietary scenarios, such as comparisons between meals, dietary patterns or national consumption statistics [4, 26, 27]. Also, the magnitude of the CO_2_e reported in this study is comparable to Bryngelsson et al 2016 [1], where similar LCA data were linked to consumption data based on national statistics from Sweden. Moreover, the daily emissions of CO_2_e was comparable to other studies based on individually assessed dietary intake using FFQ [6–8]. The dietary assessment method that was used, Meal-Q, was developed and validated by us for the LifeGene study [18, 19], and showed strong validity and reproducibility [21]. A limited number of studies have analyzed CO_2_e from self-selected diets assessed in surveys or cohort studies [6–9], but to our knowledge, this is the first study based on a Swedish population. Also, this is the first observational study looking at the relation between CO_2_e, and adherence to NNR with regards to individual nutrients. This study therefore contributes with substantial new knowledge about a diet low in GHGE based on a self-selected diet.

Differences in assumptions and methodologies between LCA studies, such as allocations and system boundaries, make comparisons between studies complicated. Product specific variations, such as use of fuel for transports, fodder for the animals, electricity mix etc. have impacts on emissions linked to each product. Moreover, the functional unit may differ, for instance, if CO_2_e is expressed for meat with or out without bones. In this study, we have taken several steps to ensure that the LCA data accurately represent food consumption in Sweden. First, the result is based on a large number of LCA studies (65 food groups) that matches the food items in dietary questionnaire [21]. Second, most LCA data used in this study have the same system boundaries and surrounding system. Thirdly, the LCA data were re-calculated taking into account weight change during cooking. Finally, we included avoidable and unavoidable waste on a household level using different values for different products [23, 24], for example, the proportion of waste is greater for fresh foods than for staple food. Although, the total estimated emissions per person may be somewhat biased, it allows us to rank individuals and make comparisons between groups of people with high and low emissions, respectively [25] and extensive sensitivity analyses show that the results are robust and did not differ when CO_2_e was divided into tertiles, quartiles or quintiles.

The cross-sectional design of the study is a limitation. Also, systematic bias may be introduced when linking data on emissions to dietary information. The FFQ is designed to capture most of the diet and the food items listed in the questionnaire is representative for main stream food habits in the population, i.e. it does not capture the whole diet [18]. Moreover, for mixed dishes, such as lasagna, we rely on standard recipes [20] and used weighted LCA data for up to three main food products or groups [21]. Also, underreporting is one of the most challenging problems with all self-reported dietary assessments, and some foods are thought to be underreported to a greater extent than others, as well as vary between groups [25]. Thus, the absolute CO_2_e is therefore underestimated in the present study as compared to if a more extensive dietary method had been used [21].

## Conclusions

In conclusion, the magnitude of the diet-related CO_2_e in the present observational study is in line with result from other studies, both scenarios and self-selected diets. Moreover, it shows that a self-selected diet low in CO_2_e provides comparable intake of nutrients associated with vegetables, fruits, meat and dairy, as a diet high in CO_2_e. Also, it shows that a diet low in CO_2_e adhere to dietary guidelines for most nutrients. This opens up for a future win-win situation between a diet low in GHGE and a nutritious diet. Our collective food choices have significant impact on global GHGE and in order to reduce climate impact from food, the consumption of meat, in particular beef, should be reduced and the consumption of plant-based foods, such as whole grains, legumes, vegetables and fruit increase.

## Additional files

Additional file 1: Table S1. Nutrient intake among women and men in the Swedish LifeGene study in 2009–10. (DOCX 14 kb)

Additional file 2: Table S2. Median nutrient intake divided by tertiles of CO_2_e adjusted for total energy intake among 5,364 men and women in the LifeGene study, 2009–10, Sweden. (DOCX 17 kb)

Additional file 3: Table S3. Median nutrient intake divided by quintiles of CO_2_e adjusted for total energy intake among 5,364 men and women in the LifeGene study, 2009–10, Sweden. (DOCX 18 kb)

## Abbreviations

μg: Microgram
BMI: Body mass index
CO_2_e: Carbon dioxide equivalents
FFQ: Food frequency questionnaire
GHGE: Greenhouse gas emission
GWP: Global warming potential
IQR: Interquartile range
kJ: Kilo joule
LCA: Life cycle assessment
Mg: Milligram
MJ: Mega joule
NNR: Nordic nutrition recommendations
