# Supplementary material for: Is a diet low in greenhouse gas emissions a nutritious diet? – Analyses of self-selected diets in the LifeGene study
Source: Arch Public Health. 2017 Apr 10;75:17. doi: 10.1186/s13690-017-0185-9 (PMC5385588; doi:10.1186/s13690-017-0185-9)
Supplement: Supplementary file 1 — Nutrient intake among women and men in the Swedish LifeGene study in 2009–10. (DOCX 14 kb) [file 13690_2017_185_MOESM1_ESM.docx]

**Additional file 1: Table S1.** Nutrient intake among women and men in the Swedish LifeGene study in 2009-10.

| **Characteristics** | | **Women**  (n=3,239) | | **Men**  (n= 2,125) | | **All**  (n= 5,364) | |
| --- | --- | --- | --- | --- | --- | --- | --- |
|  | Median | | (IQR) | Median | (IQR) | Median | (IQR) |
| Energy (kJ/d) | 7,961 | | 3,110 | 8,861 | 3,720 | 8,296 | 3,388 |
| Protein (g/d) | 73.2 | | 30.2 | 83.3 | 36.9 | 76.9 | 33.6 |
| Carbohydrates (g/d) | 214.3 | | 99.3 | 233.8 | 112.3 | 221.7 | 104.7 |
| Fat (g/d) | 63.3 | | 70.8 | 65.9 | 31.1 | 65.9 | 31.1 |
| Saturated fat (g/d) | 23.7 | | 11.9 | 27.0 | 14.3 | 24.9 | 13.1 |
| Monounsaturated fat (g/d) | 23.3 | | 11.0 | 26.2 | 12.4 | 24.3 | 11.6 |
| Polyunsaturated fat (g/d) | 10.5 | | 6.1 | 11.0 | 5.9 | 10.7 | 6.1 |
| β-carotene (µg/d) | 2,662 | | 2,600 | 2,115 | 2,137 | 2,442 | 2,437 |
| Vitamin C (mg/d) | 102.2 | | 70.0 | 90.1 | 72.8 | 97.5 | 72.2 |
| Folate (µg/d) | 300.8 | | 151.8 | 285.9 | 155.1 | 294.1 | 153.0 |
| Fiber (g/d) | 22.8 | | 13.6 | 21.6 | 13.4 | 22.3 | 13.6 |
| Vitamin B12 (µg/d) | 4.4 | | 2.5 | 5.0 | 3.0 | 4.6 | 2.7 |
| Iron (mg/d) | 12.8 | | 6.5 | 13.5 | 7.0 | 13.0 | 6.7 |
| Zinc (mg/d) | 9.9 | | 4.2 | 11.2 | 4.9 | 10.3 | 4.6 |
| Vitamin D (µg/d) | 5.5 | | 3.3 | 6.3 | 4.0 | 5.8 | 3.6 |
| Retinol (µg/d) | 393.9 | | 261.4 | 447.0 | 317.9 | 413.5 | 288.7 |
| Retinol equivalents (RE/d) | 683.8 | | 411.7 | 687.2 | 420.8 | 684.5 | 416.0 |
| Calcium (mg/d) | 906.8 | | 475.0 | 917.6 | 582.1 | 909.6 | 514.4 |
