# Supplementary material for: Is a diet low in greenhouse gas emissions a nutritious diet? – Analyses of self-selected diets in the LifeGene study
Source: Arch Public Health. 2017 Apr 10;75:17. doi: 10.1186/s13690-017-0185-9 (PMC5385588; doi:10.1186/s13690-017-0185-9)
Supplement: Supplementary file 2 — Median nutrient intake divided by tertiles of CO2e adjusted for total energy intake among 5,364 men and women in the LifeGene study, 2009–10, Sweden. (DOCX 17 kb) [file 13690_2017_185_MOESM2_ESM.docx]

**Additional file 2: Table S2.** Median nutrient intake divided by tertiles of CO_2_e adjusted for total energy intake among 5,364 men and women in the LifeGene study, 2009-10, Sweden.

|  | **kg CO_2_e/d** | | |  |  |
| --- | --- | --- | --- | --- | --- |
| **Nutrients** | 0.2 - <4.3 | 4.3 - <5.3 | 5.3 – 10.9 | P-value^1^ | P-value^2^ |
|  | Median (IQR) | Median (IQR) | Median (IQR) |  |  |
| Energy (kJ/d) | 8213(3846) | 8025(3059) | 8622(3383) | < 0.00 | < 0.00 |
| Protein (g/d) | 70.5(33.2) | 74.7(29.4) | 85.5(33.4) | < 0.00 | < 0.00 |
| Carbohydrates (g/d) | 234.2(116.5) | 215.4(97.9) | 218.3(100.6) | < 0.00 | < 0.00 |
| Fat (g/d) | 64.1(34.5) | 63.9(28.1) | 69.3(73.5) | < 0.00 | < 0.00 |
| Saturated fat (g/d) | 23.7(14.3) | 24.1(11.8) | 26.7(13.1) | < 0.00 | < 0.00 |
| Monounsaturated fat (g/d) | 23.6(12.5) | 23.5(10.8) | 25.7(11.9) | < 0.00 | < 0.00 |
| Polyunsaturated fat (g/d) | 11.0(7.1) | 10.5(5.7) | 10.7(5.5) | < 0.00 | 0.18 |
| β-carotene (µg /d) | 2405(2572) | 2444(2450) | 2460(2299) | 0.40 | 0.20 |
| Vitamin C (mg/d) | 91.5(69.6) | 100.1(71.0) | 101.8(74.3) | < 0.00 | < 0.00 |
| Folate (µg/d)^2^ | 291.4(179.4) | 292.0(142.9) | 299.8(145.8) | 0.10 | 0.25 |
| Fiber (g/d) | 24.0(17.0) | 22.2(12.3) | 21.2(11.3) | < 0.00 | < 0.00 |
| Vitamin B12 (µg/d) | 3.8(2.4) | 4.6(2.3) | 5.6(2.8) | < 0.00 | < 0.00 |
| Iron (mg/d)^3^ | 13.0(7.8) | 12.7(6.2) | 13.5(6.7) | < 0.01 | < 0.03 |
| Zinc (mg/d) | 9.6(4.9) | 10.0(4.0) | 11.4(4.5) | < 0.00 | < 0.00 |
| Vitamin D (µg/d) | 4.9(3.5) | 5.7(3.3) | 6.8(3.7) | < 0.00 | < 0.00 |
| Retinol (µg /d) | 366.8(294.6) | 408.9(255.3) | 461.0(295.6) | < 0.00 | < 0.00 |
| Retinol equivalents (RE/d) | 655.9(436.4) | 680.9(400.8) | 717.5(422.2) | < 0.00 | < 0.00 |
| Calcium (mg/d) | 812.0(476.9) | 904.3(447.2) | 1030.3(568.7) | < 0.00 | < 0.00 |
| 1. Difference between groups | |  |  |  |  |
| 2. Trend over groups | |  |  |  |  |
