# Supplementary material for: Is a diet low in greenhouse gas emissions a nutritious diet? – Analyses of self-selected diets in the LifeGene study
Source: Arch Public Health. 2017 Apr 10;75:17. doi: 10.1186/s13690-017-0185-9 (PMC5385588; doi:10.1186/s13690-017-0185-9)
Supplement: Supplementary file 3 — Median nutrient intake divided by quintiles of CO2e adjusted for total energy intake among 5,364 men and women in the LifeGene study, 2009–10, Sweden. (DOCX 18 kb) [file 13690_2017_185_MOESM3_ESM.docx]

**Additional file 3: Table S3.** Median nutrient intake divided by quintiles of CO_2_e adjusted for total energy intake among 5,364 men and women in the LifeGene study, 2009-10, Sweden.

|  | **kg CO_2_e/d** | | | | | |  |  |
| --- | --- | --- | --- | --- | --- | --- | --- | --- |
| **Nutrients** | 0.2 - <3.8 | 3.8 - <4.5 | | 4.5 – <5.1 | 5.1 – <5.9 | 5.9 – 10.9 | P-value^1^ | P-value^2^ |
|  | Median (IQR) | Median (IQR) | | Median (IQR) | Median (IQR) | Median (IQR) |  |  |
| Energy (kJ/d) | 8489(4212) | 7717(2983) | | 8163(3023) | 8347(3248) | 8740(3385) | < 0.00 | < 0.00 |
| Protein (g/d) | 71.6(36.9) | 70.1(28.0) | | 75.0(28.2) | 79.2(31.34) | 89.2(35.1) | < 0.00 | < 0.00 |
| Carbohydrates (g/d) | 249.8(132.4) | 214.1(92.7) | | 218.7(99.9) | 216.2(100.6) | 218.1(101.2) | < 0.00 | < 0.00 |
| Fat (g/d) | 65.9(39.1) | 61.6(27.9) | | 64.1(27.7) | 67.4(29.8) | 71.0(32.1) | < 0.00 | < 0.00 |
| Saturated fat (g/d) | 24.3(15.6) | 22.8(11.7) | | 24.2(12.0) | 25.4(13.1) | 27.6(12.8) | < 0.00 | < 0.00 |
| Monounsaturated fat (g/d) | 23.9(14.2) | 22.8(10.4) | | 23.6(10.8) | 24.6(11.8) | 26.6(11.9) | < 0.00 | < 0.00 |
| Polyunsaturated fat (g/d) | 11.4(7.7) | 10.3(5.9) | | 10.5(5.6) | 10.7(5.7) | 10.7 (5.4) | < 0.00 | 0.23 |
| β-carotene (µg /d) | 2534(2821) | 2281(2376) | | 2461(2463) | 2443(2254) | 2476(2411) | 0.05 | 0.15 |
| Vitamin C (mg/d) | 91.6(71.3) | 94.1(66.0) | | 98.9(70.1) | 99.8(72.3) | 104.9(76.3) | < 0.00 | < 0.00 |
| Folate (µg/d)^2^ | 303.7(200.0) | 281.5(144.5) | | 293.9(144.1) | 294.7(144.0) | 303.3(148.3) | < 0.00 | 0.26 |
| Fiber (g/d) | 25.4(19.1) | 22.0(13.6) | | 22.2(13.5) | 21.9(11.8) | 20.8(11.4) | < 0.00 | < 0.00 |
| Vitamin B12 (µg/d) | 3.7 (2.7) | 4.1(2.1) | | 4.6(2.3) | 5.0(2.3) | 5.9(3.0) | < 0.00 | < 0.00 |
| Iron (mg/d)^3^ | 13.6(8.9) | 12.2(6.5) | | 12.8(6.1) | 13.1(6.3) | 13.7(6.2) | < 0.00 | < 0.04 |
| Zinc (mg/d) | 9.7 (5.6) | 9.4 (4.0) | | 10.0 (4.0) | 10.6(4.3) | 11.8(4.6) | < 0.00 | < 0.00 |
| Vitamin D (µg/d) | 4.8 (3.7) | 5.1(3.2) | | 5.7(3.2) | 6.2(3.3) | 7.1(3.8) | < 0.00 | < 0.00 |
| Retinol (µg /d) | 366.3(322.8) | 369.6(255.9) | | 415.8(268.1) | 434.2(264.9) | 468.8(300.8) | < 0.00 | < 0.00 |
| Retinol equivalents (RE/d) | 684.8(468.3) | 632.4(383.7) | | 687.2(407.5) | 695.6(394.9) | 730.1(426.9) | < 0.00 | < 0.00 |
| Calcium (mg/d) | 813.2(520.9) | 824.7(446.1) | | 906.7(433.7) | 975.0(487.5) | 1077.3(586.6) | < 0.00 | < 0.00 |
| 1. Difference between groups | | |  |  |  |  |  |  |
| 2. Trend over groups | | |  |  |  |  |  |  |
